# Peer Review of “Cross-Modal Sensory Boosting to Improve High-Frequency Hearing Loss: Device Development and Validation”

**DOI:** 10.2196/55554

**Published:** 2024-02-09

**Authors:** Robert Eikelboom

**Affiliations:** 1 Ear Science Institute Australia, Subiaco, WA, Australia; 2 Centre for Ear Sciences, The University of Western Australia, Nedlands, Western Australia, Australia; 3 Curtin Medical School, Curtin University, Bentley, Western Australia, Australia; 4 Department of Speech-Language Pathology and Audiology, University of Pretoria, Pretoria, South Africa

**Keywords:** audiology, hearing, high-frequency, wristband, develop, development, wearable, wearables, machine learning, phoneme, phonemes, hear, vibrotactile, vibration, vibrations, sound, sounds, hearing loss, loud noise, loud noises, noise pollution, hearing aids, hearing aid

*This is the peer-review report for “Cross-Modal Sensory Boosting to Improve High-Frequency Hearing Loss: Device Development and Validation”.*

## Round 1 Review

The authors report on an interesting study [1] in which they use a wearable device to sense high-frequency sounds. I have some specific comments below. To summarize, some essential elements are missing from the manuscript, and the manuscript needs significant editorial attention (errors, academic writing style, figures).

Introduction: I would suggest using primary references for the number of people with hearing loss (rather than Olusanya et al [2]) and for the burden of hearing loss (rather than Michels et al [3]). Regarding the risk of high-frequency hearing loss, have the authors overlooked the fact that this is commonly seen in most older adults (ie, what is attributed to aging)? This is mentioned in the second paragraph. The authors are mixing up noise-related hearing loss and age-related hearing loss (presbycusis) in the manuscript.

I do not think that Hickson et al [4] is a primary reference for limitations of hearing aids (HAs) and cochlear implants.

“The auditory cortex is activated by vibrotactile information in individuals who are hearing impaired and deaf.” This implies that the auditory cortex is only activated this way.

Middle paragraph: phonemes are extracted. How this is done should be provided here, not later in the manuscript.

Is the designation of the particular transducer important? In other words, is a larger temporal difference between the two most similar phonemes important?

“The user is then able to understand...” Isn’t this yet to be shown, or is evidence provided in the next paragraph? If so, this needs to be made clearer.

Interestingly, the microphone is placed on the wrist, a part of the body that can often be situated away from the direct line of communication between two people (eg, under a table). Were the users trained to keep their wrists up?

“...listening to an audiobook, podcast...” These are often streamed to personal headsets/earphones. Were any instructions provided in terms of volume, closeness to speakers, etc?

Participants: Normally, information about participants is provided before most of the other information in a Methodology section, particularly before, for example, tasks.

Abbreviated Profile of Hearing Aid Benefit (APHAB): I am not sure that I agree with the rationale that questions on aversiveness are not relevant. Cox and Alexander [5] write “Aversiveness of Sounds, quantifies negative reactions to environmental sounds,” and “The APHAB is a potentially valuable clinical instrument. It can be useful for quantifying the disability associated with a hearing loss and the reduction of disability that is achieved with a hearing aid.” That is, it is designed to be used before an intervention (and has been used a lot for non-HA interventions as well, eg, implants).

How was the APHAB administered?

How many male and female participants were in the study?

Using an audiogram from any mobile-based device means little guarantee of accuracy.

What was the rationale for the specifications for the audiogram?

Any reason why 16 people were recruited?

What is “(11.6),” SD? And “(13)” and “(9)”?

Figure 3: I suggest not including the values on the plot. Furthermore, “Error boundary represents standard error of the mean.” The reader has to interpret the “error boundary” as the gray area.

“...to drop at a slower, more steady pace for the remaining five weeks of the study.” Writing could be tightened up a bit, and there is a rise in scores at 3 weeks. If the response is that there is not a significant increase, then it would be good to report at what point the difference is not significant.

Regression analysis: This is OK, but the use of a paired sample *t* test could have been taken for both analyses.

On the other hand, a multinomial regression analysis could have considered the influence of age, HA user or not, or baseline APHAB scores on final APHAB scores.

I see that there were approximately equal numbers of HA and non-HA users. Was this by accident or design? It is not mentioned in the Recruitment section.

Figure 6: It is not clear which score is being reported. At 6 weeks? I suspect it means the difference between the baseline and final scores. If so, this needs to be made clear in the caption.

It appears that there was no attempt to record the listening environments of the users nor how often they used their devices.

“Participants without hearing aids benefitted the most from vibrotactile sensory substitution...” True—in fact, those with HAs did not get significant benefits.

It is always good to devote a bit of space to the limitations of the study. This is missing in this manuscript.

“Future studies will focus on quantifying the maximum benefits possible and how long improvements continue before a plateau is reached.” This is not a conclusion of the study.

Perhaps this is mentioned elsewhere, but the device is given a name; it would be good to know about the association between the authors and the manufacturer of the device.

## Round 2 Review

### General Comments

The authors appear to have responded to previous comments. However, having two different versions of the manuscript in the system has caused confusion. Having some sort of system to track changes would also have been very useful.

### Specific Comments

The authors have persisted with using Michels et al [3]; this is not a primary reference for results of noise exposure of burden of hearing loss.

I am not convinced that even an omnidirectional microphone would be optimally placed on the wrist.

“...allow them to enjoy audio based entertainment such as movies and podcasts...” was of course not tested.

The last paragraph of the Introduction reads like a conclusion, not the presentation of aims or objectives.

I am unconvinced about the rationale for removing aversiveness from the APHAB; the same can be said about the other subscales. It is not about the unpleasantness introduced by the device; otherwise, why should the APHAB be applied before an intervention such as HAs or cochlear implants (as done in this study)? It is the person’s overall aversiveness to sound. Anyway, the data were not collected, so there is little to be done.

“What was the rationale for the specifications for the audiogram?”

“This was simply a general inclusion criterion to make certain we were capturing garden-variety presbycusis.”

It would be useful for this to be mentioned.

“The authors are associated both with Stanford University and the company Neosensory, which makes this device. This information is in the paper.” Okay, but I think this should extend to more than noting the affiliation of the authors. Is a financial disclosure required?

## Abbreviations

APHAB: Abbreviated Profile of Hearing Aid Benefit
HA: hearing aid
